# Association of Dietary Sugars and Sugar-Sweetened Beverage Intake with Obesity in Korean Children and Adolescents

**DOI:** 10.3390/nu8010031

**Published:** 2016-01-08

**Authors:** Kyungho Ha, Sangwon Chung, Haeng-Shin Lee, Cho-il Kim, Hyojee Joung, Hee-Young Paik, YoonJu Song

**Affiliations:** 1 Department of Public Health Nutrition, Graduate School of Public Health, Seoul National University, Seoul 08826, Korea; j015kh@snu.ac.kr (K.H.); sangwon.chung12@gmail.com (S.C.); hjjoung@snu.ac.kr (H.J.); 2 Nutrition Policy and Promotion Team, Korea Health Industry Development Institute, Cheongju 28159, Korea; leehs@khidi.or.kr; 3 Bureau of Health Industry Promotion, Korea Health Industry Development Institute, Cheongju 28159, Korea; kimci@khidi.or.kr; 4 Department of Food and Nutrition, Seoul National University, Seoul, 08826, Korea; hypaik@snu.ac.kr; 5 Major of Food and Nutrition, School of Human Ecology, The Catholic University of Korea, Bucheon 14662, Korea

**Keywords:** dietary sugars, sugar-sweetened beverage, sugars from milk and fruits, obesity, children, adolescents, Korean

## Abstract

Few studies have examined the association between dietary sugar intake and obesity in Asian children and adolescents. We evaluated the association of dietary sugar intake and its food source with obesity in Korean children and adolescents. In this cross-sectional analysis, data were obtained from five studies conducted between 2002 and 2011. The study included 2599 children and adolescents who had completed more than three days of dietary records and had anthropometric data. Total sugar intake was higher in girls than in boys (54.3 g for girls and 46.6 g for boys, *p* < 0.0001). Sugar intake from milk and fruits was inversely associated with overweight or obesity in girls only (OR for overweight, 0.52; 95% CI, 0.32–0.84; *p* for trend = 0.0246 and OR for obesity, 0.42; 95% CI, 0.23–0.79; *p* for trend = 0.0113). Sugar-sweetened beverage (SSB) consumption was not associated with obesity in girls, while boys had lower odds ratios for obesity (OR for obesity, 0.52; 95% CI, 0.26–1.05; *p* for trend = 0.0310). These results suggest that total sugars and SSB intake in Asian children and adolescents remains relatively low and sugar intake from milk and fruits is associated with a decreased risk of overweight or obesity, especially in girls.

## 1. Introduction

Obesity in childhood and adolescence is a major public health concern worldwide. The prevalence of obesity in children and adolescents has remained high and was 16.9% for two- to 19-year-olds in the United States, 14% for two- to 15-year-olds in the UK and 12.6% for eight- to 17-year-olds in Spain [1,2,3].

In Korea, the pediatric obesity rate has increased almost two-fold in the previous decade. The prevalence of obesity in young Korean boys was 12.2% and 7.2% in girls among two- to 18-year-old subjects [4,5]. Childhood obesity is associated with increased risks of chronic diseases such as diabetes and cardiovascular diseases [6,7]. Moreover, approximately 70% of obese adolescents remain obese in adulthood [8], thus preventing and managing obesity in childhood and adolescence is important.

Although complex interactions of genetic, metabolic, cultural, environmental, socioeconomic and behavioral factors contribute to obesity, dietary factors are major determinants of childhood obesity [9]. A review paper reported that excessive energy intake, the frequency of daily meals, eating alone and snack and beverage consumption were associated with childhood obesity [10]. Along with the rapid economic development and adaptation to a Western lifestyle over the past four decades, the dietary patterns of the Korean population, especially Korean adolescents, have been gradually changing. Song *et al.* [11] reported that Korean adolescents followed a secular trend from the traditional Korean diet to a Western diet between 1998 and 2005, which is associated with obesity. One characteristic of this shift to a Western diet is higher dietary sugar intake from processed foods. Recently, a number of studies investigated the association of sugar intake with weight gain or obesity. Based on a meta-analysis of 15 cohort studies that included children, high consumption of sugar-sweetened beverages (SSBs) at baseline was associated with an increased risk of overweight or obesity at follow-up [12], but no study included Asian children. Several studies examined sugar intake in Asian children but focused on sugar intake from SSBs [13,14,15]. According to the 2010–2012 Korea National Health and Nutrition Examination Survey (KNHANES), the sugar intake of Korean children and adolescents gradually increased from 66.5 g to 69.6 g in six- to 11-year-olds and 76.5 g to 80.0 g in 12- to 18-year-olds and was highest in adolescents aged 12–18 years among all age groups [16,17]. Considering the increasing trend in sugar intake, examining the associations of pediatric obesity with dietary sugar intake in the Asian population is necessary. Thus, in this study we evaluated dietary sugars and their food sources and examined the association of sugar intake with obesity in Korean children and adolescents.

## 2. Methods

### 2.1. Study Population and Data Collection

Data were obtained from five studies conducted on Korean children and adolescents between 2002 and 2011. Inclusion criteria for the secondary data analysis were that the study assessed quantitative dietary data, which included more than three days of dietary records for children and adolescents without any diseases. All five studies used the same protocols to assess dietary intake and included anthropometric data, although outcome variables such as bone mineral density or food allergy varied among the studies. Details of the five studies have been previously described [18,19,20,21,22], and the outline of each study is given in Appendix Table A1. The initial sample included 4953 children and adolescents 9–14 years of age recruited from seven elementary schools, two middle schools and a pediatric center through the university hospital in or near the Seoul metropolitan area. Inclusion criteria were if a subject completed at least three or more days of dietary records and general demographics such as age and gender without any specific disease (*n* = 4088). Among the 4088 eligible subjects, we excluded those with missing information regarding anthropometric measurements such as height and weight (*n* = 95) and missing data for maternal education (*n* = 1255) and physical activity (*n* = 139). The final sample consisted of 2599 children and adolescents (1048 boys and 1551 girls) 9–14 years of age. This study was approved by the Institutional Review Board of Seoul National University, Republic of Korea, and written informed consent was obtained from all participants (E1501/001-010).

### 2.2. Dietary Measurement and Sugar Intake

Dietary data were collected by dietary records for 3–7 days. Each subject was instructed on how to record dietary information in person by a trained staff member and asked to complete three days or more including one weekend day. The completed dietary records were reviewed by trained staff and incomplete or unclear records were clarified with the subjects at the interview.

All nutrient intakes were calculated using the CAN-Pro 3.0 (Computer Aided Nutritional Analysis Program, the Korean Nutrition Society) or DS24 (Seoul National University in Korea) software. To evaluate the adequacy of nutrient intake, the Dietary Reference Intakes for Koreans [23] was used and energy intake was compared with age- and gender-specific Estimated Energy Requirement (EER); other nutrients were compared with the Recommended Nutrient Intake (RNI).

Daily sugar intake was calculated using a total sugar database recently established for Korean food items by Lee *et al.* [17] and expanded in this study. However, the database only contains the total sugar amount for each food item; therefore, added sugars and non-milk extrinsic sugar intake could not be calculated. Thus, total sugar intake according to food source was evaluated by grouping four major food sources: fruits, milk, processed foods and commodity-type foods. Fruits included fresh fruits, dried fruits and 100% fruit juices, and milk included only white milk (whole, low-fat and nonfat). Processed foods included all types of beverages, breads and snacks, flavored milk and yogurt, ice cream and frozen confections, sweets, jams and other similar items. Commodity-type foods were defined as non-processed foods excluding milk and fruits (which were reported separately) that include non-processed ingredients such as grains, potatoes and starches, fishes and shellfishes, seaweeds, meat, eggs, vegetables, nuts, and mushrooms.

Because SSBs are major contributors to added sugar intake, we evaluated SSB intake. SSB was defined as all types of beverages including carbonated beverages, fruit and vegetable drinks, sports drinks, flavored soy milk, sweetened tea and coffee drinks, but excluding brewed coffee/tea and 100% fruit juices.

Regarding SSB intake, subjects were classified into three groups; non-drinkers who did not consume any beverage during the study period, light drinkers who consumed <200 mL/day and moderate drinkers who consumed ≥200 mL/day. The common serving size of a beverage, 200 mL, was used as the cut-off value.

### 2.3. Anthropometric Measurements and Pediatric Obesity

Anthropometric data consisted of height and weight and were measured by trained surveyors in most of the studies (92% of subjects). Only in one study (8% of subjects) data were self-reported by subjects or their parents. The body mass index (BMI) was calculated as body weight in kilograms divided by the square of body height in meters (kg/m^2^).

Pediatric overweight or obesity were defined using national reference age- and gender-specific percentiles of BMI from the Korean Growth Chart (2007); overweight if 85th percentile ≤ BMI < 95th percentile, obesity if ≥95th percentile or BMI ≥ 25 kg/m^2^. Subjects in which these percentiles overlapped with BMI within the overweight range but more than 25 kg/m^2^ were considered obese.

### 2.4. Confounding Variables

Confounding variables included basic characteristics such as age and gender and socioeconomic characteristics such as maternal educational level. Among lifestyle factors, physical activity levels were obtained.

Due to distinct differences in physical growth, we stratified all analyses according to gender (boys and girls). Because data used in this study were pooled from five studies, the study number was used as a covariate. In addition, each study had different variables and categories. Maternal education was divided into three categories: middle school or less, high school and college or more. Physical activity was measured differently in each study (time per day or number of sessions per week, or a combination of both). We categorized the population by physical activity as either physically active or not physically active. We defined “physically active” as those participating in moderate or vigorous physical activity for at least 20 min per day, or three days out of the last seven days [4].

### 2.5. Statistical Analyses

All statistical analyses were conducted using Statistical Analysis System (SAS version 9.3, SAS Institute, Cary, NC, USA). All *p*-values were two-sided and *p* < 0.05 was considered to indicate statistical significance.

Demographic and anthropometric variables were expressed as means ± standard deviation for continuous variables and percentage (%) for categorical variables. Continuous variables were tested across genders using the generalized linear model (GLM) and categorical variables were tested using the chi-square test.

Mean daily nutrient intake and sugar intake from food sources were estimated using GLM and expressed as adjusted mean ± standard error. To better identify the pattern of sugar intake, we also categorized the samples by age group. The two groups were split between primary (9–11 years of age) and secondary (12–14 years of age) school students, which is an important demographic distinction to make. We then estimated their percent contribution of sugar intake. Sugar intake from milk and fruits and processed foods by each gender were energy-adjusted using the residual method. A multinomial logistic regression analysis was performed to estimate odds ratios (ORs) and 95% confidence intervals (CI) for pediatric overweight and obesity across quartile groups and *p* for trend was also calculated with the lowest quartile set as the reference. Association of SSB with pediatric overweight and obesity was also analyzed using a multinomial logistic regression. In the multinomial logistic regression model, overweight and obesity were used as ordered outcomes, and odds ratios were estimated for each outcome on the same model.

## 3. Results

### 3.1. General Characteristics of Study Subjects

A total of 2599 Korean children and adolescents were included in our study. Mean age was 11.4 ± 1.8 years, and 40% were boys and 60% girls. Table 1 presents the general characteristics of subjects according to gender. Height, weight and BMI were significantly higher in boys than in girls (*p* < 0.0001 for all variables). In particular, boys were more frequently overweight (15.0%) than girls (13.0%). Furthermore, boys (13.9%) were significantly more frequently obese than girls (7.7%; *p* < 0.0001). Maternal educational level in girls was generally higher than that in boys, but the difference was not significant. Boys tended to have significantly higher physical activity level than girls (*p* < 0.0001).

**Table 1:** General characteristics of study subjects according to gender and age group.

	Boys (*n* = 1048)			Girls (*n* = 1551)			*p*-Value
	9–11 Years (*n* = 422)	12–14 Years (*n* = 626)	*p*-Value	9–11 Years (*n* = 950)	12–14 Years (*n* = 601)	*p*-Value	
Age (year) (Mean ± SD)	9.9 ± 0.7	13.1 ± 0.7	<0.0001	9.9 ± 0.8	13.1 ± 0.7	<0.0001	<0.0001
Height (cm) (Mean ± SD)	142.1 ± 7.1	162.6 ± 8.3	<0.0001	141.4 ± 8.0	157.4 ± 5.9	<0.0001	<0.0001
Weight (kg) (Mean ± SD)	39.8 ± 9.1	56.3 ± 12.0	<0.0001	36.9 ± 8.2	50.9 ± 9.6	<0.0001	<0.0001
BMI (kg/m^2^) ^1^ (Mean ± SD)	19.5 ± 3.3	21.2 ± 3.6	<0.0001	18.3 ± 2.9	20.5 ± 3.3	<0.0001	<0.0001
Weight status ^2^ (*n*, (%))			<0.0001			0.6486	<0.0001
Normal	311 (73.7)	492 (78.6)		759 (79.9)	477 (79.4)		
Overweight	70 (16.6)	29 (4.6)		122 (12.8)	73 (12.2)		
Obesity	41 (9.7)	105 (16.8)		69 (7.3)	51 (8.5)		
Maternal education (%)			<0.0001			<0.0001	0.4759
Middle school or less	2.6	9.6		2.8	10.2		
High school	30.3	55.4		39.6	53.7		
College or more	67.1	35.0		57.6	36.1		
Physical activity ^3^ (%)			0.0001			<0.0001	< 00001
No	35.8	47.6		48.7	68.1		
Yes	64.2	52.4		51.3	32.0		

^1^ BMI (Body Mass Index); ^2^ Overweight was defined as 85th–95th percentile for age, gender-specific BMI; obesity was defined as ≥95th percentile or BMI ≥ 25; ^3^ Physical activity was defined as “physically active” if subjects participated in moderate or vigorous physical activity for at least 20 min per day, or three days out of the last seven days; ^4^ All values for continuous variables were tested using a generalized linear model (GLM), and all values for categorical variables were evaluated using the chi-square test.

### 3.2. Nutrients and Sugar Intake

Mean daily nutrient intake and sugar intake from food sources according to gender and age group are shown in Table 2. The energy intake was 1806.0 ± 19.1 kcal in boys and 1631.9 ± 16.9 kcal in girls (*p* < 0.0001). When comparing energy intake to EER, the mean percent of EER in all groups was less than 100% and significantly lower for boys (78.8% for 9–11 years of age and 78.7% for 12–14 years of age) than for girls (85.2% for 9–11 years of age and 84.1% for 12–14 years of age; *p* < 0.0001). When comparing vitamin and mineral intake to RNI, boys showed generally higher percent RNI than girls for most nutrients, such as thiamin, niacin and iron. In addition, the nine- to 11-year-old group had a higher percent of RNI than the 12- to 14-year-old group. The percent of RNI ranged from 80% to 120%; however, the most inadequate nutrient was calcium, the percent of RNI for which was 63.9% for nine- to 11-year-olds and 49.3% for 12- to 14-year-olds in boys and 58.6% for nine- to 11-year-olds and 52.8% for 12- to 14- year-olds in girls. Conversely, the most adequate nutrients were thiamin and iron and the percent of RNI for thiamin was 143.5% for nine- to 11-year-olds and 143.8% for 12- to 14-year-olds. The values for iron were 166.6% for nine- to 11-year-olds and 112.3% for 12- to 14-year-olds in boys.

Total sugar intake in all subjects was 51.4 ± 25.0 g and the average intake in girls was 54.3 ± 0.8 g, which was significantly higher than that in boys (46.6 ± 0.9 g) after adjusting for confounding variables including energy intake. The percent of energy from total sugars was 12.5% in girls and 10.8% in boys and differed significantly according to gender.

The major food source of sugar intake in all subjects was processed foods (including beverages, breads and snacks, sweets and others). The mean sugar intake from processed foods in girls was 34.9 ± 0.6 g and was significantly greater than that in boys (27.9 ± 0.7 g). Sugar intake from processed foods was significantly higher in 12- to 14-year-olds (25.6 ± 2.6 g) than in 9- to 11-year-olds (38.0 ± 2.2 g; *p* = 0.0060). With the exception of processed foods, fruits were the major food source that contributed to total sugar intake in all groups. Sugar intake from fruits was significantly higher in girls (8.8 ± 0.4 g) than boys (7.5 ± 0.4 g; *p* = 0.0081), whereas sugar intake from milk was slightly higher in boys (6.3 ± 0.2 g) than girls (5.8 ± 0.2 g; *p* = 0.0586).

**Table 2:** Mean daily nutrient intake and sugar intake from food sources according to gender and age group.

	Boys (*n* = 1048)			Girls (*n* = 1551)			*p*-Value
	9–11 Years (*n* = 422)	12–14 Years (*n* = 626)	*p*-Value	9–11 Years (*n* = 950)	12–14 Years (*n* = 601)	*p*-Value	
Energy (kcal/day) (Mean ± SE)	1695.0 ± 72.8	1948.3 ± 61.9	0.0409	1575.5 ± 35.5	1681.9 ± 41.2	0.1124	<0.0001
Carbohydrate (g/day) (Mean ± SE)	258.6 ± 4.6	258.8 ± 3.9	0.9830	238.1 ± 2.4	236.6 ± 2.8	0.7399	0.0356
Protein (g/day) (Mean ± SE)	73.3 ± 1.9	71.0 ± 1.6	0.4697	60.9 ± 1.0	64.3 ± 1.2	0.0810	0.3124
Fat (g/day) (Mean ± SE)	57.4 ± 1.8	58.7 ± 1.5	0.6687	50.6 ± 0.9	48.3 ± 1.1	0.1826	0.9476
Percent of EER ^1^ (%)	78.8	78.7	0.9863	85.2	84.1	0.7606	<0.0001
Percent of RNI ^2^ (%)							
Thiamin	143.5	143.8	0.9944	127.7	110.2	0.0189	<0.0001
Riboflavin	103.0	78.4	0.0080	112.0	85.3	0.0002	<0.0001
Niacin	131.2	110.5	0.0864	113.1	101.8	0.0988	<0.0001
Vitamin C	83.9	66.5	0.1512	84.3	65.3	0.0465	0.0673
Calcium	63.9	49.3	0.0602	58.6	52.8	0.1590	0.1339
Phosphorus	94.2	102.3	0.3103	93.3	100.1	0.1525	0.9329
Iron	166.6	112.3	0.4549	113.7	92.6	0.3463	0.0013
Total Sugars (g/day) (Mean ± SE)	43.3 ± 3.0	56.7 ± 2.6	0.0096	52.9 ± 1.7	50.2 ± 1.9	0.3792	<0.0001
Total Sugars (% of Energy)	9.6	11.8	0.0401	12.9	12.0	0.2036	<0.0001
Sugar intake by food sources (g/day) (Mean ± SE)							
From milk	6.4 ± 0.9	5.8 ± 0.7	0.6726	5.8 ± 0.5	5.5 ± 0.5	0.7516	0.0586
From fruits	6.4 ± 1.5	7.7 ± 1.2	0.6064	9.0 ± 0.9	7.7 ± 1.1	0.4552	0.0081
From processed foods	25.6 ± 2.6	38.0 ± 2.2	0.0060	33.2 ± 1.4	32.3 ± 1.6	0.7249	<0.0001
From commodity type foods	4.8 ± 0.4	5.2 ± 0.3	0.5797	4.9 ± 0.2	4.6 ± 0.3	0.5524	0.9221

^1^ EER (Estimated Energy Requirement); ^2^ RNI (Recommended Nutrient Intake); ^3^ All values were tested using a generalized linear model (GLM) after adjusting for study number, maternal education, physical activity, and energy intake, with the exception of energy intake models.

### 3.3. Association between Dietary Sugar Intake and Obesity

Table 3 shows the multivariate adjusted ORs and 95% CIs for pediatric overweight and obesity across quartiles of dietary sugar intake based on food sources. After adjusting for confounding variables, in girls, sugars from milk and fruits were significantly associated with a lower odds of being overweight (OR for highest quartile, 0.52; 95% CI, 0.32–0.84; *p* for trend = 0.0246) and obese (OR for highest quartile, 0.42; 95% CI, 0.23–0.79; *p* for trend = 0.0113). However, no significant association was observed in boys. Moreover, sugars from processed foods were not associated with pediatric overweight or obesity in any group.

**Table 3:** Multivariate adjusted odds ratios (ORs) and 95% confidence intervals (CIs) for pediatric overweight and obesity across quartiles of dietary sugar intake based on food sources.

	Quartiles of Dietary Sugars				
	Q1	Q2	Q3	Q4	*p* for Trend
	Sugar Intake from Milk and Fruits (g/Day) ^1^				
Boys (*n* = 1048)					
Sugar intake (Mean ± SE)	3.2 ± 0.4	8.6 ± 0.4	16.0 ± 0.4	31.6 ± 0.4	
Normal	1.00				
Overweight ^2^	1.00	1.10 (0.52–2.35)	1.11 (0.52–2.37)	1.29 (0.61–2.74)	0.4617
Obesity ^2^	1.00	0.98 (0.60–1.60)	0.81 (0.48–1.38)	0.71 (0.40–1.26)	0.1952
Girls (*n* = 1551)					
Sugar intake	4.2 ± 0.4	10.7 ± 0.4	17.8 ± 0.4	34.6 ± 0.4	
Normal	1.00				
Overweight	1.00	0.61 (0.39–0.95)	0.67 (0.43–1.04)	**0.52 (0.32–0.84)**	**0.0246**
Obesity	1.00	0.69 (0.40–1.16)	0.73 (0.42–1.25)	**0.42 (0.23–0.79)**	**0.0113**
	**Sugar Intake from Processed Foods (g/day) ^1^**				
Boys (*n* = 1048)					
Sugar intake	15.1 ± 0.5	24.9 ± 0.5	34.6 ± 0.6	54.0 ± 0.5	
Normal	1.00				
Overweight	1.00	0.88 (0.45–1.72)	1.34 (0.73–2.46)	1.14 (0.61–2.14)	0.4678
Obesity	1.00	0.83 (0.52–1.33)	0.54 (0.32–0.92)	0.70 (0.42–1.15)	0.0894
Girls (*n* = 1551)					
Sugar intake	15.4 ± 0.5	25.0 ± 0.5	34.6 ± 0.5	53.1 ± 0.5	
Normal	1.00				
Overweight	1.00	1.00 (0.65–1.54)	0.87 (0.56–1.35)	1.08 (0.69–1.67)	0.8152
Obesity	1.00	0.86 (0.51–1.46)	0.80 (0.47–1.37)	0.92 (0.53–1.58)	0.7673

^1^ Sugar intake from milk and fruits or processed foods was energy-adjusted using the residual method and categorized into quartiles; ^2^ Overweight was defined as 85th–95th percentile for age, gender-specific BMI; obesity was defined as ≥95th percentile or BMI ≥ 25; ^3^ A multinomial logistic regression analysis was used to test associations between dietary sugars and obesity after adjusting for age, study number, maternal education, and physical activity.

### 3.4. Association between Sugar-Sweetened Beverage Intake and Obesity

Table 4 shows the multivariate-adjusted ORs and 95% CIs for pediatric overweight and obesity across SSB intake. Among subjects, 43% were non-drinkers whereas 10.7% of boys and 7.7% of girls consumed 200 mL or more per day. In terms of the percent of energy from SSB, girls consumed 6.0% of their energy from SSB, which was higher than that in boys (5.8%).

After adjusting for confounding variables, boys who consumed more than 200 mL per day had 48% lower odds of being obese (OR, 0.52; 95% CI, 0.26–1.05; *p* for trend = 0.0310) compared to boys who did not drink SSBs; there was a statistically significant trend, but the OR was not statistically significant. However, no significant association was observed in girls.

**Table 4:** Multivariate-adjusted odds ratios (ORs) and 95% confidence intervals (CIs) for pediatric overweight and obesity across sugar-sweetened beverage intake.

	Sugar-Sweetened Beverage (SSB) Intake (mL/Day) ^1^			
	Non-Drinker	<200 mL/Day	≥200 mL/Day	*p* for Trend ^3^
Boys (*n* = 1048)				
*n* (%)	461 (44.0)	475 (45.3)	112 (10.7)	
SSB intake (Mean ± SE)	0	87.8 ± 3.2	301.7 ± 5.2	
SSB (% of energy)	0	2.1	5.8	
Normal	1.00			
Overweight ^2^	1.00	1.29 (0.81–2.07)	0.70 (0.29–1.69)	0.5150
Obesity ^2^	1.00	0.61 (0.42–0.90)	0.52 (0.26–1.05)	**0.0310**
Girls (*n* = 1551)				
*n* (%)	647 (41.7)	785 (50.6)	119 (7.7)	
SSB intake	0	74.4 ± 2.1	260.9 ± 4.0	
SSB (% of energy)	0	2.0	6.0	
Normal	1.00			
Overweight	1.00	0.95 (0.69–1.30)	1.00 (0.53–1.87)	0.9097
Obesity	1.00	1.26 (0.84–1.89)	1.36 (0.62–2.97)	0.3325

^1^ SSB intake was categorized into two groups: those who drank SSB < 200 mL and SSB ≥ 200 mL per day among SSB drinkers; ^2^ Overweight was defined as 85th–95th percentile for age, gender-specific BMI; obesity was defined as ≥95th percentile or BMI ≥ 25; ^3^ A multinomial logistic regression analysis was used to test associations between SSB and obesity after adjusting for age, study number, energy intake, maternal education, and physical activity.

## 4. Discussion

In the large sample study of 2599 Korean children and adolescents 9–14 years of age, we found that total dietary sugar intake and its contribution to total energy intake remained relatively low, although the sugar intake from processed foods comprised more than half of the percent of total sugar intake. Regarding dietary sugar intake based on food sources, only sugar intake from milk and fruits was inversely associated with pediatric overweight as well as obesity among girls.

The average daily total sugar intake from three or more days of dietary records was 51.4 g in this study and was comparable with 61.3 g, the recently reported total sugar intake among six- to 11-year-old Korean children from one-day 24-h recall data in a national nutrition survey in 2008–2011 [17]. However, this level of dietary sugar is considerably lower than that of children and adolescents in Western countries. The daily total sugar intake reported was 139 g in one- to 18-year-old subjects in the United States [24], 107.1 g in 11- to 18-year-old subjects in the UK [25], and 172 g in boys 14–18 years of age in Canada [26].

SSB intake was also reported to be lower in an Asian populations compared to a Western population. In this study, the mean SSB intake was 63 mL/day, which was lower than the 127 mL/day reported in a study of Australian children and adolescents 2–16 years of age [27]. The mean energy from SSBs in the present study was 27 kcal/day, which is lower than in children and adolescents in the United States with the values of 141 kcal/day for boys and 112 kcal/day for girls among 6- to 11-year-old subjects and 273 kcal/day for boys and 171 kcal/day for girls among 12- to 19-year-old subjects [28]. Unexpectedly, sugars from processed foods were not associated with pediatric overweight or obesity. Conversely, high SSB intake was associated with the reduced OR of obesity in boys.

Reportedly, high SSB intake is associated with weight gain and obesity, and a recent meta-analysis confirmed this positive relationship; however, all of the studies were conducted in Western children [12,29]. Only a few studies investigating the association between SSB and obesity in Asian children were performed; however, the results were inconsistent.

SSBs not only contain high amounts of added sugars that lead to adverse health outcomes but also are associated with unhealthy food patterns or eating behaviors [30]. In terms of dietary patterns or eating behaviors among people who consume high amounts of SSB, Asian children and adolescents may have different dietary practices. With the rapid economic growth and adaptation to Western lifestyle, the traditional pattern has shifted to the modified pattern in which high animal foods or sweets (including SSBs) were introduced to the traditional diet [11]. According to studies conducted in Korea and China, higher consumption of SSBs was associated with higher socioeconomic status [31,32].

In terms of different dietary practices in Asia, the mixed findings regarding the relationship between SSBs and obesity can be, in part, explained. SSB drinkers consumed more total energy and showed a higher prevalence of obesity in Chinese children [14] and Korean boys 7–12 years of age [33]. Conversely, sugar intake from SSBs and snacks was not significantly associated with weight status in Japanese and Cambodian children [15].

In general, total energy intake in Asian children and adolescents is lower than in Western children and adolescents. In this study, the average percent of energy intake compared to age- and gender-specific EER was approximately 85% in girls and even less in boys (<80%).

A significant and inverse association of sugar from milk and fruits was observed only in girls. Due to the absence of an added sugars database in Korea, we were unable to evaluate added or free sugars intake. Alternatively, we evaluated dietary sugar intake from various food sources; only sugars from milk and fruits showed a meaningful influence on reduced weight. Milk and fruits have protective effects on weight gain and obesity in children [34,35,36]. However, in our study we showed that even sugar intake from milk and fruits has a positive association with obesity, especially in girls, and should be considered when developing strategies for weight management in Korea. Korean girls seek a slim body image and most have tried dieting at least once [37,38]. Due to the current increased focus on dietary sugar intake, fruits are often misunderstood as a source of a high level of simple sugars.

As children and adolescents grow, they can develop undesirable dietary behaviors in this complex food environment. Among subjects less than 19 years of age, mean energy from added sugars linearly increased with age in United States children [39] and total sugar intake in Korean children showed a similar increasing trend with age [17]. In addition, Canadian adolescents 9–18 years of age consumed more added sugars; conversely, children 1–8 years of age consumed sugars from natural food sources [40]. Lee *et al.* [33] reported that SSB intake was inversely associated with milk, fruit and vegetable intake in Korean children and adolescents. Our additional analysis showed lower sugar intake from milk and fruits and higher sugar intake from processed foods in subjects 12–14 years of age compared to those 9–11 years of age. Therefore, monitoring not only dietary sugar intake but also the food source is important, and unsweetened milk instead of SSB should be encouraged in this age group along with fresh fruits. In addition, effective nutrition education or policies should be implemented to maintain low sugar intake in Korean children and adolescents.

This study had several limitations. First, the sample was pooled from five previous studies. Although we collected all core information, such as three or more days of dietary records and anthropometric measurements, discrepancies between studies may exist. To minimize this bias, the study number was used as a covariate in all analyses. Second, under-reporting of dietary intake could have occurred because the study subjects were children that completed three or more days of dietary records. Third, we obtained dietary data from three to seven days of food records. Most dietary data included non-consecutive days with at least one weekend day. However 0.1% of subjects did not report for at least one weekend day. This may be a possible source of limitation in estimating usual intake for this population. However, since no study was reported to estimate usual intake for dietary sugars using multiple days of food records, this study provides a meaningful addition to report the dietary sugar intake for Korean children and adolescents.Fourth, SSB intake data in this study were from the previous decade rather than current one because the five original studies were conducted from 2002 to 2011. Due to the rapid growth in the SSB market in Asian countries [13,41], the current SSB intake is expected to increase considerably; thus, the association with obesity will differ. Finally, this study was cross-sectional in design, making it difficult to determine a causal relationship between dietary sugar intake and the risk of obesity.

Despite these limitations, this study had several strengths. To the best of our knowledge, this is the first study to estimate dietary sugar intake and the food sources thereof using multiple days of dietary records in a large sample of Asian children and adolescents. Since daily sugar intake or SSB intake was calculated individually from three or more days of dietary records, it should be representative of the usual intake.

## 5. Conclusions

In conclusion, dietary sugars or SSB intake in Asian children and adolescents is lower than in Western children and adolescents. Sugars from milk and fruits correlate with a decreased risk of obesity in Asian girls from this cross-sectional study. Dietary sugar intake should be monitored continually and further prospective studies examining the relationship between dietary sugar intake and obesity are necessary.

## Appendix

**Table A1:** Outlines of the five studies used in this analysis.

	Study 1	Study 2	Study 3	Study 4	Study 5
Sample size (*n*, (%))	1294 (49.8)	447 (17.2)	219 (8.4)	525 (20.2)	114 (4.4)
Year of study	2002–2003	2006	2008	2008–2009	2011
IRB number ^1^	Not applicable	2006-12-06-28	2008-03-28-53	2008-10-07-68	SPIRB-11-033
Days of dietary records (Mean ± SD)	3.0 ± 0.1	6.0 ± 0.2	3.0 ± 0.0	3.0 ± 0.0	3.0 ± 0.0
Age (year) (Mean ± SD)	11.6 ± 1.7	13.4 ± 0.6	10.2 ± 1.0	10.0 ± 0.9	10.0 ± 1.2
Age group (*n*, (%))					
9–11 years old	571 (44.1)	0 (0)	204 (93.2)	498 (94.9)	99 (86.8)
12–14 years old	723 (55.9)	447 (100)	15 (6.9)	27 (5.1)	15 (13.2)
Gender (*n*, (%))					
Boys	649 (50.2)	244 (54.6)	108 (49.3)	0 (0)	47 (41.2)
Girls	645 (49.9)	203 (45.4)	111 (50.7)	525 (100)	67 (58.8)

^1^ IRB (Institutional Review Board).
